# Binding of HIV-1 gp41-Directed Neutralizing and Non-Neutralizing Fragment Antibody Binding Domain (Fab) and Single Chain Variable Fragment (ScFv) Antibodies to the Ectodomain of gp41 in the Pre-Hairpin and Six-Helix Bundle Conformations

**DOI:** 10.1371/journal.pone.0104683

**Published:** 2014-08-08

**Authors:** John M. Louis, Annie Aniana, Katheryn Lohith, Jane M. Sayer, Julien Roche, Carole A. Bewley, G. Marius Clore

**Affiliations:** 1 Laboratories of Chemical Physics, National Institute of Diabetes and Digestive and Kidney Diseases, National Institutes of Health, Bethesda, Maryland, United States of America; 2 Bioorganic Chemistry, National Institute of Diabetes and Digestive and Kidney Diseases, National Institutes of Health, Bethesda, Maryland, United States of America; Chinese Academy of Medical Sciences, China

## Abstract

We previously reported a series of antibodies, in fragment antigen binding domain (Fab) formats, selected from a human non-immune phage library, directed against the internal trimeric coiled-coil of the N-heptad repeat (N-HR) of HIV-1 gp41. Broadly neutralizing antibodies from that series bind to both the fully exposed N-HR trimer, representing the pre-hairpin intermediate state of gp41, and to partially-exposed N-HR helices within the context of the gp41 six-helix bundle. While the affinities of the Fabs for pre-hairpin intermediate mimetics vary by only 2 to 20-fold between neutralizing and non-neutralizing antibodies, differences in inhibition of viral entry exceed three orders of magnitude. Here we compare the binding of neutralizing (8066) and non-neutralizing (8062) antibodies, differing in only four positions within the CDR-H2 binding loop, in Fab and single chain variable fragment (ScFv) formats, to several pre-hairpin intermediate and six-helix bundle constructs of gp41. Residues 56 and 58 of the mini-antibodies are shown to be crucial for neutralization activity. There is a large differential (≥150-fold) in binding affinity between neutralizing and non-neutralizing antibodies to the six-helix bundle of gp41 and binding to the six-helix bundle does not involve displacement of the outer C-terminal helices of the bundle. The binding stoichiometry is one six-helix bundle to one Fab or three ScFvs. We postulate that neutralization by the 8066 antibody is achieved by binding to a continuum of states along the fusion pathway from the pre-hairpin intermediate all the way to the formation of the six-helix bundle, but prior to irreversible fusion between viral and cellular membranes.

## Introduction

**Figure 1:**
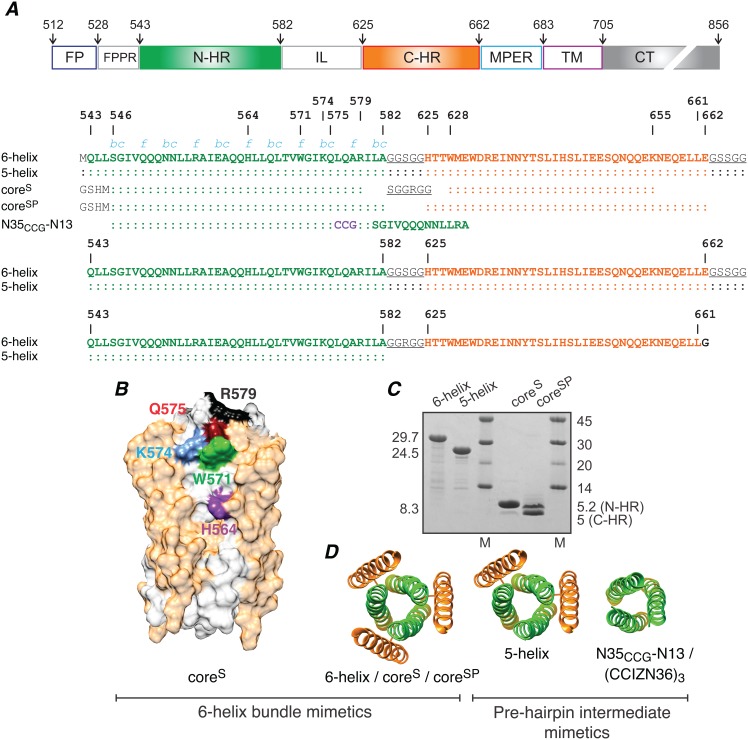
Engineered mimetics of the pre-hairpin intermediate and post-fusion six-helix bundle of HIV-1 gp41. (*A*) Domain organization of HIV-1 gp41 and sequences of the six-helix bundle (6-helix, core^S^ and core^SP^) and pre-hairpin (5-helix and N35_CCG_-N13) mimetics. (FP, fusion protein; FPPR, fusion peptide proximal region; N-HR, N-heptad repeat; IL, immune-dominant linker; C-HR, C-heptad repeat; MPER, membrane proximal external region; TM, transmembrane region; CT, intraviral C-terminal domain.) Three N35_CCG_-N13 chains are linked covalently via intermolecular disulfide bridges (CCG, shown in purple) to form a stable helical trimer [19]. N-HR, C-HR and linker residues are shown in green, orange and black (underlined), respectively. Numbering of N-HR and C-HR regions is according to their location in Env from HIV-1 (strain HXB2). Positions in the helical wheel (blue italic) of N-HR residues that are solvent accessible in the six-helix bundle conformation are indicated. (*B*) Interactions of Fab8066 with N-HR residues in the context of the six-helix bundle construct core^S^ mapped by Ala scanning mutagenesis and immunoblotting [22]. The core^S^ trimer is shown as a surface representation with N-HR and C-HR regions of gp41 in white and light orange, respectively. N-HR surface accessible residues (H564, W571, K574 and Q575) identified as sites of interaction with Fab8066 are shown in distinct colors. (*C*) Relative migration on SDS-PAGE of the gp41 mimetics used in this study. Molecular weights of constructs and markers (M) are indicated in kDa. N-HR and C-HR denote peptides which assemble to form the six-helix bundle conformation of core^SP^. (*D*) Ribbon representations of the gp41 constructs. 5-helix, CCIZN36 and N35_CCG_-N13 are pre-hairpin intermediate mimetics with one or more exposed N-HR helices, that are otherwise partially shielded in the six-helix bundle. The three N-HR peptide chains in N35_CCG_-N13 and CCIZN36 are stabilized as disulfide-linked trimers by fusion with either a 13-residue repeat of the N-HR [19] or an N-terminal isoleucine zipper segment [37], respectively. 6-helix and 5-helix are single chain polypeptides with the N-HR (N) and C-HR (C) regions connected by a six-residue linker in the order N-C-N-C-N-C and N-C-N-C-N, respectively [18]. Core^S^ and core^SP^ also form a six-helix bundle but as a trimer consisting of 3 hairpin (N-linker-C) peptides and a hexamer consisting of 3 N-HR and 3 C-HR peptides, respectively (see panel A).

The surface envelope (Env) glycoproteins of HIV-1, gp120 and gp41, mediate fusion of the viral and cell membranes [1]. The initial events in the fusion process involve the binding of CD4 and the chemokine co-receptor to gp120 triggering a series of conformational changes in both gp120 and gp41 that culminate in fusion of the viral and cell membranes [2], [3], [4], [5], [6], [7]. Early steps in this process, representing a possible “activated” state of gp120/gp41, have recently been visualized by crystallography and cryo-electron microscopy of a soluble cleaved HIV-1 Env trimer [8], [9]. In these Env structures, gp41 is in a pre-fusion state: the trimeric coiled-coil N-heptad repeat (N-HR, residues 542–591) and the C-terminal heptad repeat (C-HR, residues 623–663) do not interact with one another and both structural elements are solvent accessible. This structure approximates the postulated pre-hairpin intermediate in which the viral and cell membranes are bridged via the C- and N-termini of gp41, respectively [4], [10], [11]. The final conformational rearrangement occurs further along the fusion pathway and involves the formation of a six-helix bundle, the so-called fusogenic/post-fusogenic state, in which the N-HR trimeric helical coiled-coil is surrounded by three C-HR helices [12], [13], [14], [15], [16]. The six-helix bundle brings the viral and cell membranes into contact with one another which eventually leads to fusion [11]. Various constructs have been devised to mimic both the pre-hairpin intermediate [17], [18], [19] and six-helix bundle conformations of gp41 (Figs. 1A and D) [12], [16], [18].

The N-HR trimer in the pre-hairpin intermediate state of gp41 is transiently accessible (neutralization half-life ∼20 min) during the course of fusion [4], [10], [20] and is the target of several fusion inhibitors, including various monoclonal antibodies [20], [21], [22], [23], [24], [25], [26], [27], peptides derived from the C-HR of gp41 [10], [28], [29], [30], and a peptide derived from the N-HR that inhibits trimerization of the N-HR of gp41 by sequestering the N-HR into heterotrimers [31]. Interestingly the latter potentiates the neutralization activity of N-HR targeted antibodies (and even rescues neutralization activity) by prolonging the temporal window for inhibition [32].

In a series of papers [20], [21], [22] we described a set of monoclonal antibodies selected from the HuCal Gold human non-immune phage library [33], [34] by panning against the chimeric construct N_CCG_-gp41 [17] which presents the N-HR as a stable, helical disulfide-linked trimer that extends in helical phase from the six-helix bundle of gp41. Panned antibodies that recognized either six-helix bundle or N-HR trimer constructs were found to be non-neutralizing. Only antibodies that recognized both the six-helix bundle and N-HR trimer constructs were neutralizing. Subsequent affinity maturation by targeted diversification of the CDR-H2 loop resulted in a fragment antigen binding domain (Fab), known as Fab8066, that was highly potent and broadly neutralizing across a wide range of primary HIV-1 isolates and laboratory-adapted HIV-1 strains [22].

**Figure 2:**
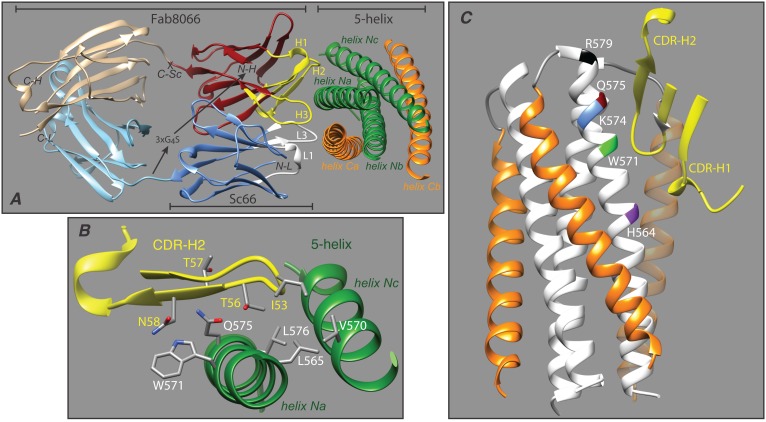
Interaction of Fab8066 with 5-helix and design of the corresponding ScFv Sc66. (*A*) Overall interaction of Fab8066 with 5-helix, (*B*) detailed view of the interaction of the CDR-H2 loop of Fab8066 (yellow) with the two exposed N-HR helices (green) of 5-helix, and (*C*) interaction of the CDR-H1 and CDR-H2 loops of Fab8066 (yellow) with two N-HR helices (white) and one C-HR helix (orange) of 5-helix. The addition of a third C-HR helix (transparent orange) to 5-helix to complete the six-helix bundle would result in steric clash with the CDR-H1 and CDR-H2 loops. Color coding in panels A and B is as follows: N-HR and C-HR helices of 5-helix are shown in green and orange, respectively; the CDR heavy and light chain loops of Fab8066 are shown in yellow and white, respectively; the remainder of the heavy and light variable domains are shown in dark red and blue, respectively; the light and heavy constant domains of Fab8066 are shown in pink and light blue, respectively. In panel C, the three N-HR helices are shown in white and residues mapped by alanine scanning mutagenesis of a six-helix bundle construct as the epitope for binding Fab8066 [20], [21], [22], [23], [24], [25], [26], [27], are indicated on one of the N-HR helices (helix *Na*). Also shown in panel A is the design employed to construct the corresponding ScFv by linking the C-terminus of the light chain variable domain (blue) to the N-terminus of the heavy-chain variable domain (dark red) via a 15-amino acid linker (3×GGGGS). The coordinates are taken from PDB IDs 3MA9 (Fab8066/5-helix complex [35]) and 1SZT (core^S^ trimer [16]).

Crystal structures of Fab8066 and a non-neutralizing Fab (Fab8062) from the same affinity matured series differing in only 4 positions in the CDR-H2 loop, complexed to two mimetics of the pre-haipin intermediate (constructs comprising the N-HR timer surrounded by either two or no C-HR helices resulting in 1∶1 and 1∶3 mimetic:Fab complexes) revealed only subtle structural differences between complexes with the neutralizing and non-neutralizing Fabs [35], [36]. Moreover, despite differences in neutralizing activity of three or more orders of magnitude in Env-pseudotyped virus neutralization assays [22], the binding affinities of the neutralizing and non-neutralizing Fabs to various pre-hairpin intermediate mimetics differ by little more than an order of magnitude [35], [36]. This suggested to us that the ability to bind to the six-helix bundle, in addition to the exposed N-HR trimer of the pre-hairpin intermediate, may be critical to the neutralization activity of this particular series of Fabs. Interestingly, alanine scanning mutagenesis and Western blot analysis showed that this series of Fabs targets a structurally contiguous epitope on the N-HR that is solvent accessible and located in a shallow groove between two C-HR helices in the six-helix bundle (Fig. 1B) [20], [22]. The same residues of the N-HR also comprise part of the binding site in the pre-hairpin intermediate mimetics (Fig. 2A, B) [35], [36]. However, if the Fabs were to bind to the six-helix bundle in the same mode as seen in the crystal structures with the pre-hairpin intermediate mimetics, there would be steric clash and atomic overlap between the CDR-H1 and CDR-H2 loops of the Fab and one of the C-HR helices of the six-helix bundle (Fig. 2C). The simplest hypothesis for these observations is that binding of this series of Fabs to the six-helix bundle involves displacement of one of the C-HR helices.

Here we investigate the binding of the 8066 and 8062 monoclonal antibodies, as well as of various point mutants within the CDR-H2 loop of 8066, in Fab and single chain-variable fragment (ScFv) formats to mimetics of the pre-hairpin intermediate and six-helix bundle conformation. We show that binding to the six-helix bundle does not involve displacement or fraying of a C-HR helix, indicating that these antibodies must interact somewhat differently with the six-helix bundle and the pre-hairpin intermediate mimetics, although they share a common epitope. Further, we show that there are large differences in binding affinity of neutralizing and non-neutralizing antibodies within this series to the six-helix bundle.

## Results and Discussion

### Binding affinities of Fab8066 and Fab8062 to pre-hairpin intermediate mimetics

Both the neutralizing Fab8066 and the non-neutralizing Fab8062 recognize an epitope at the C-terminal end of the N-HR timer of gp41 comprising a hydrophobic pocket formed at the interface of two N-HR helices (Figs. 2A and B) [35], [36]. The relative binding affinities for Fab8066 and Fab8062 to various pre-hairpin intermediate mimetics, as determined by isothermal titration calorimetry (ITC), differ by factors ranging from 20-fold to only 2-fold. In the case of 5-helix, a single chain pre-hairpin mimetic that presents a single antibody binding site in which the N-HR trimer is surrounded by two C-HR helices, the binding affinities differ by ∼20 (≤10 vs ∼200 nM) [35]; for N35_CCG_-N13, an N-HR trimer construct stabilized by disulfide bridges [19] that presents three antibody binding sites, the binding affinities differ by ∼8 (∼30 vs ∼250 nM; Fig. S1 in File S1); and for CCIZN36, an N-HR trimer construct stabilized by a disulfide-linked leucine zipper [37], which also presents three antibody binding sites, the binding affinities differ by a factor of only 2 (∼5 versus ∼10 nM) [36]. This variation might be attributable to differences in conformational flexibility of the N-HR trimer between the various pre-hairpin mimetics that may facilitate accommodation of the conformational differences within the CDR-H2 binding loops of Fab8066 and Fab8062 [36].

### Binding affinities of ScFv variants of Fab8066 and Fab8062 to 5-helix

To further investigate which residues within the CDR-H2 are important for binding target antigen and to ascertain the impact of size on biophysical and biological properties, we engineered ScFv variants (∼26 kDa) of Fab8066 and Fab8062 (∼49 kDa), denoted as Sc66 and Sc62, respectively, as well as a series of four single substitution mutants of Sc66 (I53L, T56F, T57A and N58V) that match the residue variation in the CDR-H2 region of Sc62 (Fig. 2B and Fig. S2 in File S1). The binding affinities of these six ScFvs to 5-helix are in the low nM range and cover a range that differs by about an order of magnitude (Table 1 and Fig. S1 in File S1): Sc66 and Sc62 differ by a factor of 7, Sc66_I53L_ and Sc66_T57A_ are comparable to the parent Sc66, Sc66_N58V_ is about three-fold weaker than Sc66, and Sc66_T56F_ is about 13 and 2-fold weaker than Sc66 and Sc62, respectively. (Note that the N58V and T56F mutations each remove a hydrogen bond to the carboxyamide group of Gln575 of one of the N-HR helices of 5-helix [35]; cf. Fig. 2B). These binding results are consistent with those obtained with the parent Fabs.

**Table 1:** Comparison of equilibrium dissociation constants for the binding of ScFvs to 5-helix determined by ITC and HIV-1 neutralization potency in an Env-pseudotyped neutralization assay.

Antibody	ITC *K*_D_ (nM)^a^	HIV-1 neutralization IC_50_ (nM)			
		HXB2	JRCSF	SF162	89.6
Fab8066^b^	<<10	82±29	230±160	510±150	380±110
Sc66	2.0±1.5	50±15	460±160	350±140	470±280
Sc62^c^	14.0±4.2	≥6000			
Sc66_I53L_	3.8±1.9	14±4	160±30	150±40	230±30
Sc66_T56F_	27.0±6.5	≥6000			
Sc66_T57A_	3.0±1.0	71±13			
Sc66_N58V_	6.4±2.3	≥6000			

a ITC was carried out at 28°C in 10 mM Tris-HCl, pH 7.6, 150 mM NaCl.

b The neutralization IC_50_ values for Fab8066, taken from ref. [22], are provided for comparison. The *K*_D_ for binding to 5-helix, also determined by ITC under the same conditions used for the ScFvs here, is from ref. [35].

c Sc62 is derived from the parental Fab8062 and has four mutations in the CDR-H2 loop relative to Fab8066/Sc66: I53L, T56F, T57A and N58V.

### HIV-1 neutralization by the ScFv variants of Fab8066 and 8062

The neutralization properties of the ScFv variants were compared to those of the parent Fabs using an Env-pseudotyped neutralization assay [20], [38], [39] (Table 1). The neutralization potencies (IC_50_) of Sc66 and Fab8066 are comparable for several HIV-1 strains (Table 1). Although Sc62, unlike Fab8062 [22], displays weak neutralization activity, it is more than 2 orders of magnitude less potent than Sc66. The Sc66_T56F_ and Sc66_N58V_ mutations result in a reduction in neutralization potency by more than two orders of magnitude. Interestingly at the highest concentration employed in the assay (20 µM), Sc62, Sc66_N58V_ and Sc62_T56F_, inhibit fusion by close to 100%, while Fab8062 displays no inhibitory effect even at this high concentration [22]. In contrast the Sc66_T57A_ mutation only reduces neutralization activity by a factor of 2, while the Sc66_I53L_ mutation increases neutralization activity 2–3 fold. Thus, while there is an approximate correlation between binding affinity to the pre-hairpin mimetic 5-helix and neutralization activity, the differential is far greater (more than two orders of magnitude) for neutralization than binding (approximately an order of magnitude).

### Binding of Fabs and ScFvs to the six-helix bundle mimetic core^s^

Given that all the neutralizing Fabs in our series were shown to bind to both pre-hairpin intermediate and six-helix bundle mimetics of gp41 by Western blotting, we decided to investigate in more detail the binding of our Fabs and ScFv variants to three six-helix bundle mimetics (Figs. 1C, D). These include core^S^, a trimer in which each subunit comprises a single N-HR and C-HR connected by a six-residue linker [16]; core^SP^, a hexamer assembled from three N-HR and three C-HR peptides [12]; and finally 6-helix, a single chain in which the N-HR and C-HR helices are sequentially joined together by five six-residue linkers (in the order N-C-N-C-N-C, where N and C represent the N-HR and C-HR helices, respectively) [18].

**Figure 3:**
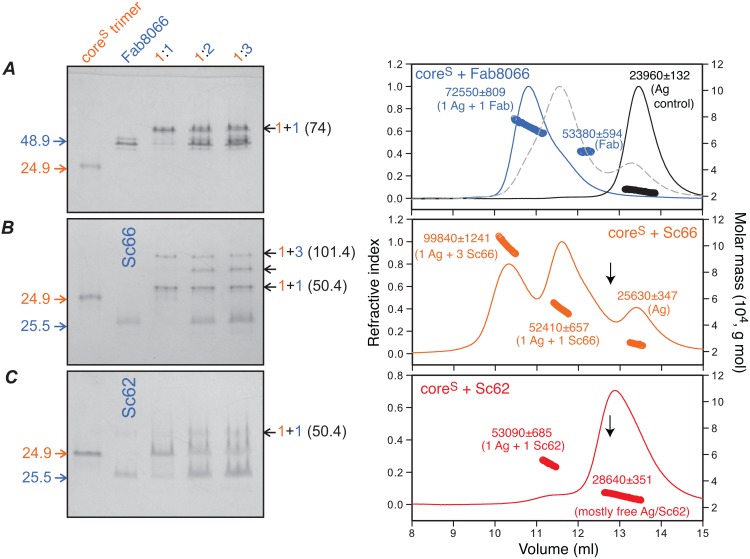
Native-PAGE band-shift and SEC-MALS analyses of core^S^-antibody complexes. Interaction of (*A*) Fab8066, (*B*) Sc66 and (*C*) Sc62 with the six-helix bundle core^S^ antigen (Ag). Left panels: 10 µM core^S^ trimer mixed with Fab or ScFv (shown above the lanes) in molar ratios of 1∶1, 1∶2 and 1∶3 were subjected to 20% homogeneous native-PAGE. Core^S^ and antibody are color coded orange and blue, respectively. Calculated molecular weights of Fab8066, Sc66, Sc62, core^S^ and their complexes are indicated in kDa. Right panels: Protein mixtures (total of 200 µg) at a trimer (core^S^) to antibody ratio of 1∶1 were subjected to SEC-MALS. Experimental average masses and compositions are indicated. Also shown in panel A (right) are the SEC-MALS traces for core^S^ alone (black), corresponding to a trimer (of calculated mass 3×8284 g/mol), and a 1∶1 mixture of core^S^ and Fab8062 (dashed gray) which shows no evidence of complex formation. The black arrows in panels B and C (right) indicate the retention volume of Sc66 (see Figure S2C in File S1). In panel C (right), the major peak corresponds to uncomplexed ScFv and core^S^ which co-elute, while the minor peak represents a barely detectable amount of 1∶1 Sc62:core^S^ complex. Observed masses (g/mol) for free Fab8066, Fab8062 and Sc66 are 47060±559, 48560±629 and 27630±774, respectively.

ITC measurements on the interactions of Fabs and ScFvs with the three six-helix bundle mimetics displayed negligible thermal responses. We therefore resorted to optimize a method to monitor antigen-antibody binding for these various constructs by native polyacrylamide gel electrophoresis (native-PAGE) and analytical size-exclusion chromatography with in-line multiangle light scattering, refractive index and UV detectors (SEC-MALS) (Fig. 3).

On native-PAGE core^S^ migrates between Fab8066 and Sc66 (Fig. 3, left panels), and elutes as a trimer on SEC-MALS (Fig. 3A, right panel, black trace). When mixed at a 1∶1 ratio of 10 µM core^S^ trimer and 10 µM Fab8066, nearly all the core^S^ and Fab8066 shift in position to a slower migrating complex (marked 1+1) on native-PAGE above the Fab8066 band (Fig. 3A, left panel). The same 1∶1 mixture elutes as a single peak on SEC-MALS corresponding to a combined mass of 1 core^S^ timer+1 Fab8066 (∼73 kDa), with a very small shoulder, corresponding to free Fab8066 (∼53 kDa). Addition of more Fab8066 at 1∶2 and 1∶3 core^S^:Fab ratios does not result in the binding of additional Fab8066 molecules to core^S^, as evidenced by the presence of an excess Fab8066 band in the native-PAGE lanes (Fig. 3A, left panel). A similar 1∶1 mixture of core^S^ and Fab8062 barely shows any shift on native-PAGE and elutes on SEC-MALS mainly in free form with masses of ∼52 and 26 kDa, corresponding to Fab8062 and core^S^, respectively (Fig. 3A, right panel, dashed trace).

In the case of Sc66, both 1∶1 and 1∶3 core^S^:Sc66 complexes are apparent on both native-PAGE (Fig. 3B, left panel) and SEC-MALS (Fig. 3B, right panel). That the peaks seen in SEC-MALS correspond to the same compositions as observed by native-PAGE was verified by subjecting peak fractions from the SEC-MALS column to native and SDS-PAGE (Fig. S3 in File S1). In contrast, Sc62 binds only very weakly to core^S^ and only a very small amount of 1∶1 complex is apparent by either native-PAGE (Fig. 3C, left) or SEC-MALS (Fig. 3C, right). Similar results are seen with the ScFv mutants: the behavior of the neutralizing mutants Sc66_I53L_ and Sc66_T57A_ is very similar to Sc66, while the non-neutralizing mutants Sc66_T56F_ and Sc66_N58V_ behave like Sc62 (Fig. S4 in File S1).

### Binding of Fabs and ScFvs to the six-helix bundle mimetics core^sp^ and 6-helix

**Figure 4:**
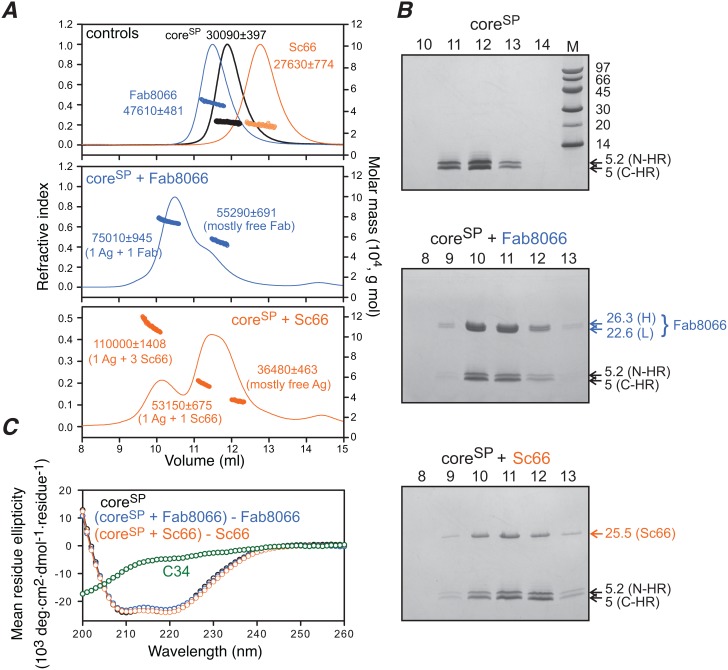
Mass, SDS-PAGE and CD analyses of core^SP^-antibody complexes. (*A*) SEC-MALS of core^SP^ (black), Fab8066 (blue) and Sc66 (orange) (top) and of the core^SP^-Fab8066 (middle) and core^SP^-Sc66 (bottom) complexes (mixed at a molar ratio of 1∶1 six-helix bundle to antibody). Average masses and compositions are indicated next to the peaks. (*B*) SDS-PAGE of peak fractions (numbering of lanes corresponds to elution volume) collected from SEC-MALS confirm the composition of the peaks indicated on the SEC-MALS traces in panel A. Top, core^SP^ alone; middle, core^SP^+Fab8066 (H and L denote heavy and light chains, respectively); bottom, core^SP^+Sc66. (*C*) CD of core^SP^ alone (black); a 1∶1 mixture of (core^SP^+Fab8066) minus Fab8066 alone (blue); a 1∶1 mixture of (core^SP^+Sc66) minus Sc66 alone (orange); and the C34 peptide (green, corresponding to the C-HR) alone. The CD data indicate that there is no change in helicity of core^SP^ when complexed to either Fab8066 or Sc66.

**Figure 5:**
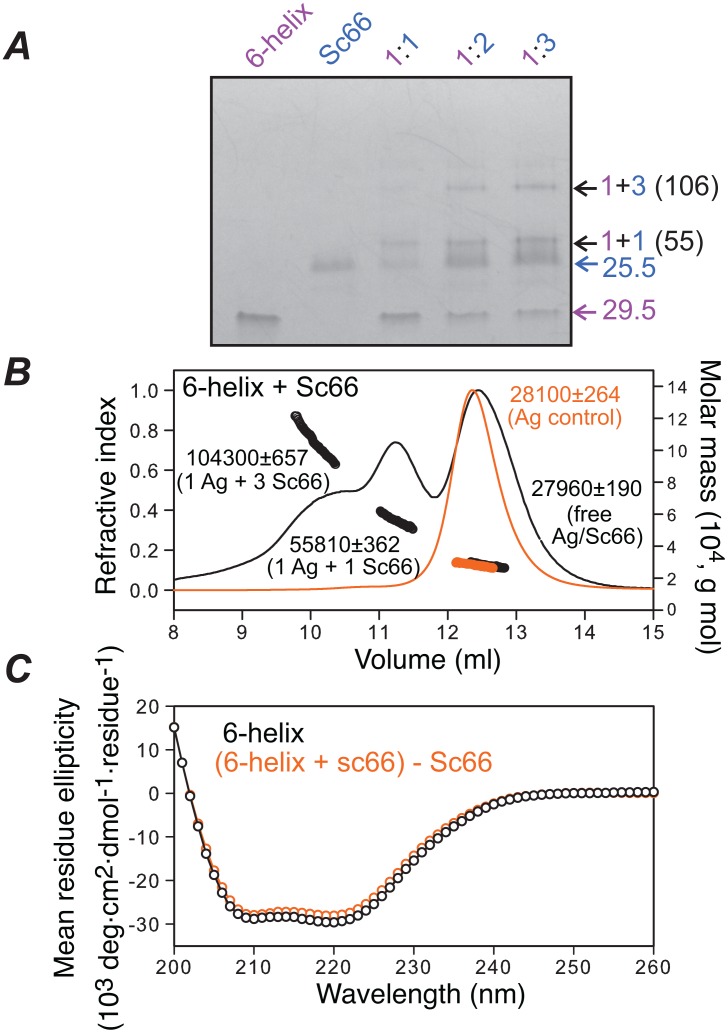
Native-PAGE, SEC-MALS and CD analysis of 6-helix-antibody complexes. (*A*) Native-PAGE in the presence of increasing molar ratios of Sc66 to 6-helix. 6-helix to Sc66 ratios are shown above the respective lanes and the observed stoichiometry of the complexes (purple, 6-helix; blue, Sc66) and expected molecular weights (kDa) are indicated. (*B*) SEC-MALS for 6-helix alone (orange) and a 1∶1 mixture of 6-helix with Sc66 (black). Average compositions and masses are indicated next to the peaks. (*C*) CD spectra of 6-helix alone (black) and a 1∶2 mixture of (6 helix+Sc66) minus Sc66 alone (orange). The CD data indicate that there is no change in helicity of 6-helix upon complexation with Sc66.

**Figure 6:**
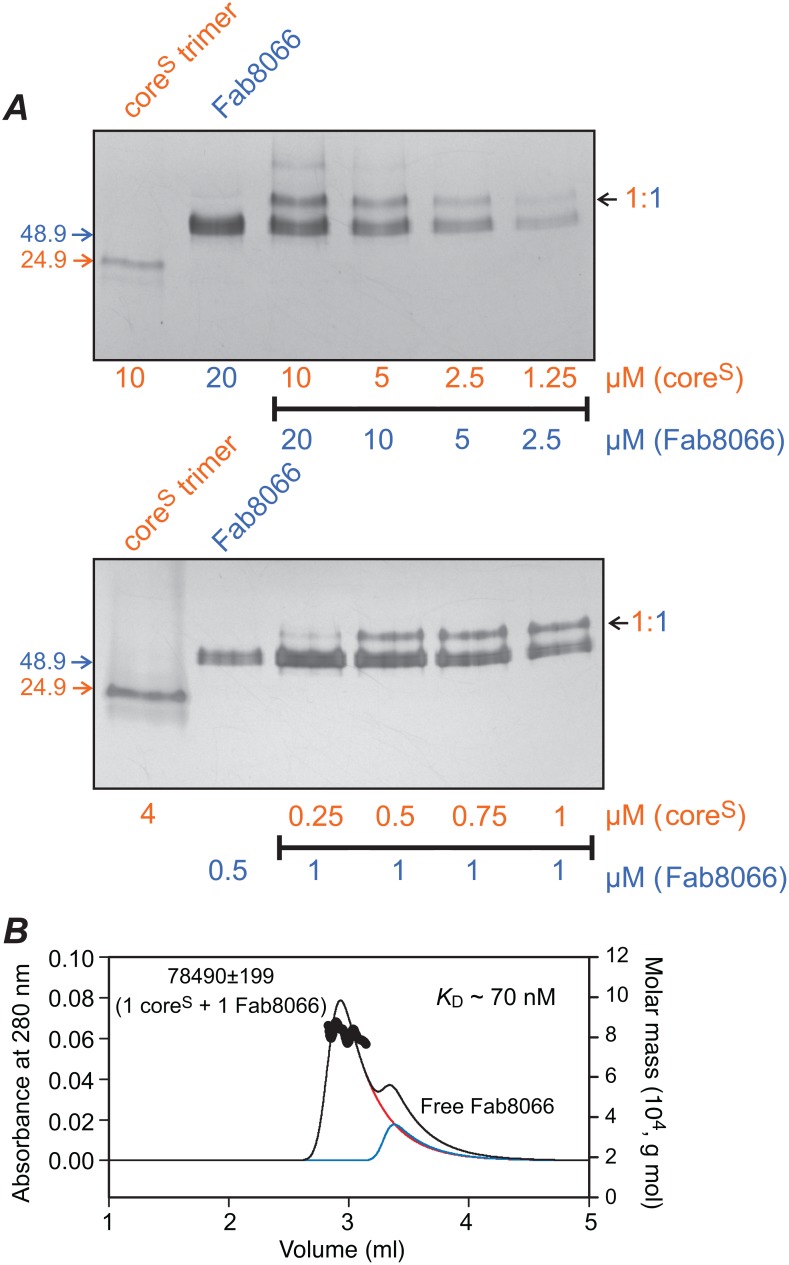
Determining the upper limit of affinity for the binding of Fab8066 to core^S^ by native-PAGE and an approximate *K*_D_ by SEC-MALS. (*A*) Decreasing concentrations (10 to 1.25 µM) of core^S^ trimer mixed with a 2-fold molar excess of Fab8066 and subjected to native-PAGE with Coomassie staining (upper panel), and decreasing concentrations (1 to 0.25 µM) of core^S^ trimer mixed with a constant 1 µM concentration of Fab8066, visualized by silver staining. Core^S^ and Fab8066 are color coded orange and blue, respectively. (*B*) Injection of 3 µg core^S^ mixed with 6 µg Fab8066 on a BioSep-SEC-S 2000 column (0.46×30 cm) at a flow-rate of 0.35 ml/min equilibrated in 10 mM Tris-HCl, pH 7.6, 150 mM NaCl (buffer A). The elution profile (black) is shown superimposed on deconvoluted peaks for the major (∼85%) core^S^-Fab8066 complex (red) and free Fab8066 (blue). The measured mass of the complex is shown beside the peak. A *K*_D_ of ∼70 nM was estimated on the basis of the calculated concentration of the complex and free Fab. Deconvolution of the SEC-MALS profile was carried out using the program PeakFit (Seasolve Software, Inc. Framingham, MA).

The binding of Fab8066 and Sc66 to core^S^ does not allow one to distinguish whether binding occurs directly to the surface accessible region of the N-HR helices between the surrounding C-HR helices in the six-helix bundle conformation, or whether binding involves displacement (or fraying) of one or more C-HR helices in the six-helix bundle permitting interaction with the resulting fully exposed epitope on the trimeric N-HR coiled coil. To this end we investigated the binding of Fab8066 and Sc66 to core^SP^ (Fig. 4) and 6-helix (Fig. 5). In the case of core^SP^, displacement of the C-HR would result in dissociation of the C-HR helix into free solution (where the free C-HR peptide would adopt a largely random coil structure with a very weak CD helical signature; cf. Fig. 4C and Fig. S4C in File S1), while for 6-helix only a single C-HR could be displaced without unraveling the protein.

The SEC-MALS profiles observed for 1∶1 mixtures (10 µM of each component) of Fab8066 and Sc66 with core^SP^ are similar to those obtained with core^S^. Specifically, a 1∶1 complex is observed for the interaction of Fab8066 with core^SP^ (Fig. 4A, middle panel) while 1∶1 and 1∶3 antigen:ScFv complexes are observed for the interaction of Sc66 with core^SP^ (Fig. 4A, bottom panel). SDS-PAGE of the elution fractions indicate that the 1∶1 and 1∶3 antigen:antibody complexes contain N-HR and C-HR peptides together with heavy and light chains for Fab8066 (Fig. 4B, middle panel) and a single chain for Sc66 (Fig. 4B, bottom panel). In addition, circular dichroism (CD) of 1∶1 mixtures of core^SP^ and Fab8066 or Sc66 indicates that the helicity of core^SP^ remains unchanged upon complexation with antibodies (Fig. 4C and Fig. S5A and S5B in File S1). Likewise, native-PAGE (Fig. 5A) and SEC-MALS (Fig. 5B) of a 1∶1 mixture (10 µM of each component) of Sc66 and 6-helix reveal the presence of 1∶1 and 1∶3 antigen:ScFv complexes with no change in helicity of 6-helix upon complexation with Sc66 as monitored by CD (Fig. 5C and Fig. S5D in File S1). Thus, one can conclude unambiguously that no displacement of the C-HR is involved upon binding of Fab8066 and Sc66 to 6-helix bundle mimetics, and therefore the mode of interaction of these antibodies with the 6-helix bundle mimetics must be slightly different from that observed by crystallography with pre-hairpin intermediate mimetics [35], [36] to avoid steric clash between the CDR-H1 and CDR-H2 loops of the antibodies and one of the C-HR helices (Fig. 2C).

### Estimating the binding affinity of Fab8066 to Core^s^ by native-PAGE and SEC-MALS

Since we were unable to successfully use ITC to quantitatively determine the binding affinities of our neutralizing antibodies in either Fab or ScFv formats to the six-helix bundle mimetics, we used native-PAGE band-shift analysis to obtain a semi-quantitative upper limit for the strength of the interaction of Fab8066 with core^S^. Fab8066 rather than Sc66 was used in this assay since the larger size of Fab8066 improves detectability on gel staining and Fab8066 exclusively forms a 1∶1 complex with core^S^ rather than the mixture of 1∶1 and 1∶3 antigen:Fab complexes observed with Sc66. In the first set of native-PAGE band-shift experiments we prepared 1∶2 mixtures of core^S^ trimer with Fab8066 in 10 mM Tris-HCl, pH 7.6, 150 mM NaCl at Fab concentrations ranging from 10 to 1.25 µM. As the limit of detectability with Coomassie staining was reached at 1.25 µM of the core^S^-Fab8066 1∶1 complex (Fig. 6A, top), silver staining was used to provide improved detection at lower concentrations. A set of dilutions (Fig. 6A, bottom) was made in which Fab8066 at a constant concentration of 1 µM was titrated by addition of core^S^ to give final concentrations ranging from 1 µM to 0.25 µM core^S^. The increase in band intensity of the 1∶1 complex, accompanied by a gradual decrease in intensity of free Fab8066, in going from 0.25 to 1 µM core^S^ shows that complex formation is accompanied by depletion of Fab8066. At the lowest concentration of core^S^ only a small amount of the 1∶1 complex can be detected. This serves to set an upper limit of *K*_D_≤250 nM for the binding of Fab8066 to core^S^. By way of contrast, a lower limit of *K*_D_>>10 µM for the binding of Fab8062 and Sc62 to core^S^ can be estimated from the SEC-MALS and native-PAGE data shown in Figs. 3 A and C, respectively.

To further refine our *K*_D_ estimate for the interaction of Fab8066 with core^S^ we made use of SEC-MALS, injecting very small amounts of core^S^ (3 µg) and Fab8066 (6 µg) (Fig. 6B). Deconvolution of the SEC-MALS profile together with concentration estimations from the known volumes of the peaks yielded an approximate *K*_D_ of 70 nM.

### Concluding remarks

The HIV-1 broadly neutralizing Fabs selected from the HuCal Gold human non-immune phage library by panning and subsequent affinity maturation (by targeted diversification of the CDR-H2 loop) against N_CCG_-gp41, a chimera comprising the fully exposed N-HR trimer linked in helical phase to a disulfide-linked six-helix bundle of gp41, are characterized by binding to both the fully exposed N-HR trimer and the six-helix bundle [20], [22]. Fabs from this series that only bound to either the N-HR trimer or the six-helix bundle were found to be non-neutralizing. In this light we sought to characterize in more detail the binding of two monoclonal antibodies from this series (in Fab and ScFv formats) to mimetics of the pre-hairpin intermediate and six-helix bundle conformations of gp41. One of the antibodies, Fab8066 (Sc66), is broadly neutralizing, while the second, Fab8062 (Sc62), differing in only four positions within the CDR-H2 loop, is non-neutralizing [22]. The difference in neutralization potency between these two antibodies ranges from two to over three orders of magnitude, depending on format (ScFv versus Fab), yet the difference in binding affinity to three different pre-hairpin intermediate mimetics ranges from only 2 to 20-fold. Further single point mutations in the CDR-H2 of Sc66, at positions where the CDR-H2s of Sc66 and Sc62 differ, show that the residues at positions 56 and 58 are critical for neutralization activity, but have only a relatively small effect on binding affinity to the pre-hairpin intermediate mimetic 5-helix (Table 1). SEC-MALS and native-PAGE analysis indicate that a significantly larger difference (≥150-fold) in binding affinity of neutralizing (Fab8066) and non-neutralizing (Fab8062) Fabs is obtained with core^S^ (and expected to be the same for core^SP^ and 6-helix). Moreover, SEC-MALS, CD and SDS-PAGE demonstrate conclusively that no displacement (or local fraying) of the C-HR helix (helices) occurs upon Fab binding to the six-helix bundle mimetics. Previous scanning mutagenesis and Western blotting analysis showed that the epitope for the binding of Fab8066 to the six-helix bundle involves surface-exposed residues at the C-terminus of the N-HR located in a groove between two adjacent C-HR helices [20], [22]. This epitope overlaps with that observed in crystal structures of Fab8066 and Fab8062 with pre-hairpin intermediate mimetics [35], [36]. Since the introduction of a C-HR helix would result in steric clash with the CDR-H1 and CDR-H2 loops in the latter complexes (Fig. 2C), these results imply that the details of the interaction of the Fabs with solvent accessible N-HR residues in the six-helix bundle must be sufficiently different from those observed when the N-HR trimer is fully exposed (as in the pre-hairpin intermediate mimetics) to circumvent steric clash with a C-HR helix.

What is the relevance of the ability of neutralizing Fabs within our series to bind to both pre-hairpin intermediate mimetics and six-helix bundles of gp41? The time window at which the neutralizing Fabs act is very similar to that of peptides derived from the C-HR of gp41, as well as a peptide (N36^Mut(eg)^) engineered from the N-HR to not interact with the C-HR, thereby inhibiting gp41 trimerization by forming heterotrimers with one or two gp41 N-HR helices [20], [22], [32]. The C-HR derived inhibitory peptides can only target the fully exposed N-HR trimer of the pre-hairpin intermediate, and thus both the Fabs and the N36^Mut(e,g)^ peptide must also target this state. In addition, the N36^Mut(e,g)^ peptide could also potentially act at subsequent stages in the fusion process in which the six-helix bundle is either partially formed (the pre-bundle state [40]) or fully formed but before irreversible membrane fusion actually takes place. The observation that our series of neutralizing Fabs can also bind to the six-helix bundle suggest that the neutralizing Fabs can target six-helix bundle conformations prior to actual fusion in addition to the pre-hairpin intermediate, and that the ability to target a continuum of states from the pre-hairpin intermediate to the six-helix bundle prolongs the time window over which these Fabs can inhibit fusion, thereby increasing their potency and neutralization properties.

## Materials and Methods

### *E. coli* plasmid constructs

Previously reported plasmid constructs used in this study are the six-helix bundle constructs 6-helix [18] and core^S^ [16], [21], and the pre-hairpin mimetics 5-helix [18], and N35_CCG_-N13 [19]. Constructs used for the first time are the ScFvs Sc66, Sc62, Sc66_I53L_, Sc66_T56F_, Sc66_T57A_, Sc66_N58V_, and the six-helix bundle construct core^SP^. The strategy for linking the light and heavy variable regions as a single chain to generate Sc66 from Fab8066 is shown in Fig. 2A [41] and its amino acid sequence in Figure S1A. DNA inserts were cloned either in pET11a or pET15 vectors between Nde1/BamH1 and Nco1/BamH1 sites, respectively. Site-directed mutagenesis was carried out using the Quik-Change mutagenesis kit (Agilent Technologies) on Sc66 DNA (template) to derive Sc62, Sc66_I53L_, Sc66_T56F_, Sc66_T57A_ and Sc66_N58V_ constructs. DNA sequences and purified proteins were verified by DNA sequencing and electrospray ionization mass spectrometry (ESI-MS), respectively.

### Protein purification and folding

*E. coli* BL21(DE3) bearing the appropriate plasmid vector were grown in Luria-Bertani medium and induced for expression at an A_600_ ∼0.7 for 4 hrs. All of the expressed proteins used in this study accumulated in the insoluble fraction [19]. After isolating the insoluble fraction (∼80% purity), the various gp41 constructs were further purified on size-exclusion Superdex-200 or 75 columns under denaturing conditions. They were finally subjected to reverse-phase HPLC using a water/TFA and acetonitrile/TFA gradient. The desired amount of protein was dialyzed against 50 mM sodium formate, pH 3, concentrated (8–10 mg/ml) and stored. When needed, an aliquot of the protein was diluted in 10 mM Tris-HCl, pH 7.6, 150 mM NaCl (buffer A), dialyzed against the same buffer and concentrated.

Core^SP^ was derived from a construct comprising the N-HR (residues 546–582) and C-HR (residues 625–662) of gp41 separated by a spacer SGLVPRGSGG (Fig. 1A). Thrombin cleavage was carried out resulting in the separation of the N-HR and C-HR regions prior to purification. These peptides were assembled by dialysis from 6 M guanidine hydrochloride into 10 mM Tris-HCl, pH 7.6, 150 mM NaCl (buffer A) to form a six-helix bundle complex but without a spacer connecting the N-HR and C-HR regions as in core^S^, and fractionated as a complex on a Superdex-75 column in buffer A. The pre-hairpin intermediate mimetic N35_CCG_-N13 trimer was prepared as described [21]. ESI-MS of N35_CCG_-N13 showed a trimer mass of 22634 Da close to the calculated value of 22638 Da (3×7546). Experiments with N35_CCG_-N13 were carried out in 50 mM sodium acetate, pH 5.2 because of its insolubility above this pH.

ScFvs were folded using previously described protocols [41], [42], [43]. Upon folding from denaturing conditions, Sc66 exhibited nearly complete disulfide bridge formation (Fig. S1B in File S1). The ScFvs were finally subjected to size-exclusion chromatography on Superdex-75 in buffer A. The concentrated ScFvs (1–2 mg/ml) are stable upon storage for several months at 4°C, and retain their monomeric status, binding affinity as determined by ITC, and neutralization activity (see below). All of the purified and folded ScFvs used for various characterizations are shown in Fig. S1C in File S1. By SEC-MALS (Fig. S1D in File S1) Sc66 was shown to elute with a monomer mass of 27630±774 g/mol (calculated 25540). Sc66 mutants and Sc62 also exhibit similar elution profiles.

The theoretical masses of the various proteins used in the study in kDa are as follows: 6-helix (29,470), 5-helix (24,459), core^S^ (8,284×3), core^SP^ (N-HR: 5216, C-HR: 4975), Fab8066 (48,896) and Sc66 (25,540).

### Isothermal titration calorimetry

ITC measurements were made using a Microcal high-precision iTC200 titration calorimeter (GE Healthcare). Titrations were performed at 28°C. Protein in the sample cell was titrated either with nineteen 2-µL injections or fifteen 2.45-µL injections of titrant. ITC of Fab8066 with core^S^ or 6-helix (as titrant) and of Sc66 with 6-helix (as titrant) when performed at 28°C or 37°C in 10 mM Tris-HCl, pH 7.6, 150 mM NaCl (buffer A) yielded no thermal response. Data were analyzed using the Origin software provided with the instrument.

### Molecular mass analysis

Molecular masses were analyzed by SEC-MALS (DAWN EOS or Wyatt-925-H2HC; DAWN Heleos, Wyatt Technology Inc., Santa Barbara, CA), refractive index (Wyatt-215-TRXH; Optilab T-rEX, Wyatt Technology Inc.) and UV (Waters 2487, Waters Corporation, Milford, MA) detectors. Volumes of injection ranged from 100–150 µl. Typically 200 µg of total protein was mixed in a molar ratio of 1∶1 antigen to antibody. Individual proteins were injected as controls. The sample was centrifuged at 12,800 rpm for 4 min in an Eppendorf 5415 centrifuge and the supernatant applied to a pre-equilibrated Superdex-75 column (1.0×30 cm, GE Healthcare) at a flow rate of 0.5 ml/min at room temperature and eluted in buffer A, unless stated otherwise. Under these conditions, the eluting concentrations are expected to be 6–10 µM, near the concentrations used for native-PAGE. Molecular masses were calculated using the Astra software (version 6) provided with the instrument.

### Viral fusion assays

Assays to quantify viral entry in the presence of antibodies employed Env-pseudotyped HIV strains with host cells genetically modified to express CD4 and co-receptors CCR5 and CXCR4, as described previously [20], [38], [39]. Envs were derived from four HIV-1 subtype B laboratory-adapted strains, HXB2, SF162, JR CSF and 89.6. The IC_50_ represents the midpoint of the sigmoid dose-response curve obtained from a fit to a simple dose-response relationship (percent infection = S×100/(1+[Ab]/IC_50_) where S is a scale factor.

### Native-PAGE

Samples were mixed to achieve a final concentration of 10 µM core^S^ (as trimer, unless stated otherwise) and appropriate molar ratio of antibody as indicated. This concentration was chosen because it represents a good detection limit to differentiate between the binding of neutralizing and non-neutralizing Fabs and ScFvs. They were incubated at room temperature for 30 min and 2 µl of sample was layered for electrophoresis on a 20% homogeneous Phastgel using 1 µl/eight-lane applicators (unless stated otherwise) and native buffer strips (GE Healthcare). Gels were stained in PhastGel Blue R, destained and digitized. For detection at lower concentrations, 4 µl/six-lane applicators and PlusOne silver staining kit (GE Healthcare) were used.

### Circular dichroism

CD spectra were recorded in 10 mM Tris-HCl, pH 7.6, 150 mM NaCl (buffer A) at 20°C on a JASCO J-810 spectropolarimeter using Spectra Manager software and a 0.1 cm path length flat cell. Compositions similar to those used for band-shift and SEC-MALS analysis were used. Mean residue ellipticity was calculated using the instrument’s software.

## Supporting Information

File SI Five supporting figures. **Figure S1.** ITC analysis of Fab and ScFv binding to two pre-hairpin intermediate mimetics. Binding of (*A*) Fab8066 and (*B*) Fab8062 to the disulfide-linked trimer N35_CCG_-N13 in 50 mM sodium acetate pH 5.2 buffer (required to maintain the solubility of N35_CCG_-N13). 10 µM Fab was in the cell and N35_CCG_-N13 (∼100 **µ**M in monomer) was the titrant. (*C* to *F*) Binding of ScFvs to 5-helix in 10 mM Tris-HCl, pH 7.6, 150 mM NaCl. 5-helix (5–7 µM) in the cell was titrated with the designated ScFv at a concentration of 50–70 µM. HIV-1 neutralizing antibodies are shown in the left-hand panels and non-neutralizing on the right-hand panels. Values of the equilibrium association constant (*K*_A_) are shown in each panel. **Figure S2**. Expression, purification and folding of functional ScFvs. Sc66 and Sc62 are derived from the broadly neutralizing Fab8066 and the non-neutralizing Fab8062, respectively [S1]. (*A*) Amino acid sequence of Sc66. Sc62 differs from Sc66 at 4 positions (displayed in green) within the CDR-H2 loop. In the ScFvs, the light (blue) and heavy (orange) variable regions are separated by the 15 residue spacer sequence 3×(GGGGS) that connects the C-terminus of the light chain variable domain to the N-terminus of the heavy chain variable domain. (*B*) 20% homogeneous SDS-PAGE of Sc66 under oxidizing (lanes O) and reducing (lanes R) conditions shows that complete disulfide bridge formation can be visualized by the faster migration of oxidized Sc66 (lanes O) relative to the reduced sample (lanes R) and can therefore be monitored during ScFv folding. (*C*) 20% homogeneous SDS-PAGE of Sc66, Sc62 and four Sc66 mutants containing point mutations within the CDR-H2. Numbering of the mutations corresponds to their position in the heavy chain variable sequence of Fab8066 [S2]. Numbers beside the marker lane (M) in both gel panels denote molecular weights in kDa. (*D*) The mass of Sc66 determined by SEC-MALS corresponds to a monomer (calculated mass, 25,540). **Figure S3.** Native- and SDS-PAGE of peak fractions from SEC-MALS analysis of 1∶1 mixtures of core^S^-Fab8066 and core^S^-Sc66 complexes. Gels shown in (*A*) and (*B*) correspond to elution profiles and masses shown for core^S^-Fab8066 and core^S^-Sc66 in Figs. 3A and B, respectively, in the main text. Numbering of lanes corresponds to elution volume. **Figure S4.** Native-PAGE of core^S^-Sc66 single mutant complexes. No binding is observed with Sc66_T56F_, and relatively weak (1∶1) binding is seen with Sc66_N58V_. Shifts in the presence of mutants Sc66_I53L_ and Sc66_T57A_ closely resemble those for Sc66. Core^S^ and ScFv are color coded orange and blue, respectively. Calculated molecular weights of core^S^, ScFv and their corresponding complexes are indicated in kDa. **Figure S5**. Raw CD data for Fab8066 and Sc66 complexed to the six-helix bundles core^SP^ and 6-helix. (*A*) core^SP^+Fab8066; (*B*) core^SP^+Sc66; (*C*) the C-HR peptide C34; (*D*) 6 helix+Sc66.
(PDF)
