# Supplementary material for: Binding of HIV-1 gp41-Directed Neutralizing and Non-Neutralizing Fragment Antibody Binding Domain (Fab) and Single Chain Variable Fragment (ScFv) Antibodies to the Ectodomain of gp41 in the Pre-Hairpin and Six-Helix Bundle Conformations
Source: PLoS One. 2014 Aug 8;9(8):e104683. doi: 10.1371/journal.pone.0104683 (PMC4126735; doi:10.1371/journal.pone.0104683)
Supplement: File SI — Five supporting figures. Figure S1. ITC analysis of Fab and ScFv binding to two pre-hairpin intermediate mimetics. Binding of (A) Fab8066 and (B) Fab8062 to the disulfide-linked trimer N35CCG-N13 in 50 mM sodium acetate pH 5.2 buffer (required to maintain the solubility of N35CCG-N13). 10 µM Fab was in the cell and N35CCG-N13 (∼100 µM in monomer) was the titrant. (C to F) Binding of ScFvs to 5-helix in 10 mM Tris-HCl, pH 7.6, 150 mM NaCl. 5-helix (5–7 µM) in the cell was titrated with the designated ScFv at a concentration of 50–70 µM. HIV-1 neutralizing antibodies are shown in the left-hand panels and non-neutralizing on the right-hand panels. Values of the equilibrium association constant (K A) are shown in each panel. Figure S2. Expression, purification and folding of functional ScFvs. Sc66 and Sc62 are derived from the broadly neutralizing Fab8066 and the non-neutralizing Fab8062, respectively [S1]. (A) Amino acid sequence of Sc66. Sc62 differs from Sc66 at 4 positions (displayed in green) within the CDR-H2 loop. In the ScFvs, the light (blue) and heavy (orange) variable regions are separated by the 15 residue spacer sequence 3×(GGGGS) that connects the C-terminus of the light chain variable domain to the N-terminus of the heavy chain variable domain. (B) 20% homogeneous SDS-PAGE of Sc66 under oxidizing (lanes O) and reducing (lanes R) conditions shows that complete disulfide bridge formation can be visualized by the faster migration of oxidized Sc66 (lanes O) relative to the reduced sample (lanes R) and can therefore be monitored during ScFv folding. (C) 20% homogeneous SDS-PAGE of Sc66, Sc62 and four Sc66 mutants containing point mutations within the CDR-H2. Numbering of the mutations corresponds to their position in the heavy chain variable sequence of Fab8066 [S2]. Numbers beside the marker lane (M) in both gel panels denote molecular weights in kDa. (D) The mass of Sc66 determined by SEC-MALS corresponds to a monomer (calculated mass, 25,540). Figure S3. Na [file pone.0104683.s001.pdf]

SUPPORTING INFORMATION

Binding of HIV-1 gp41-directed neutralizing and non-neutralizing fragment antibody binding domain (Fab) and single chain variable fragment (ScFv) antibodies to the ectodomain of gp41 in the pre-hairpin and six-helix bundle conformations.

John M. Louis[1,*], Annie Aniana[1], Katheryn Lohith[2], Jane M. Sayer[1], Julien Roche[1], Carole A. Bewley[2] and G. Marius Clore[1,*]

From the [1]Laboratories of Chemical Physics and [2]Bioorganic Chemistry, National Institute of Diabetes and Digestive and Kidney Diseases, National Institutes of Health, Bethesda, Maryland 20892-0520

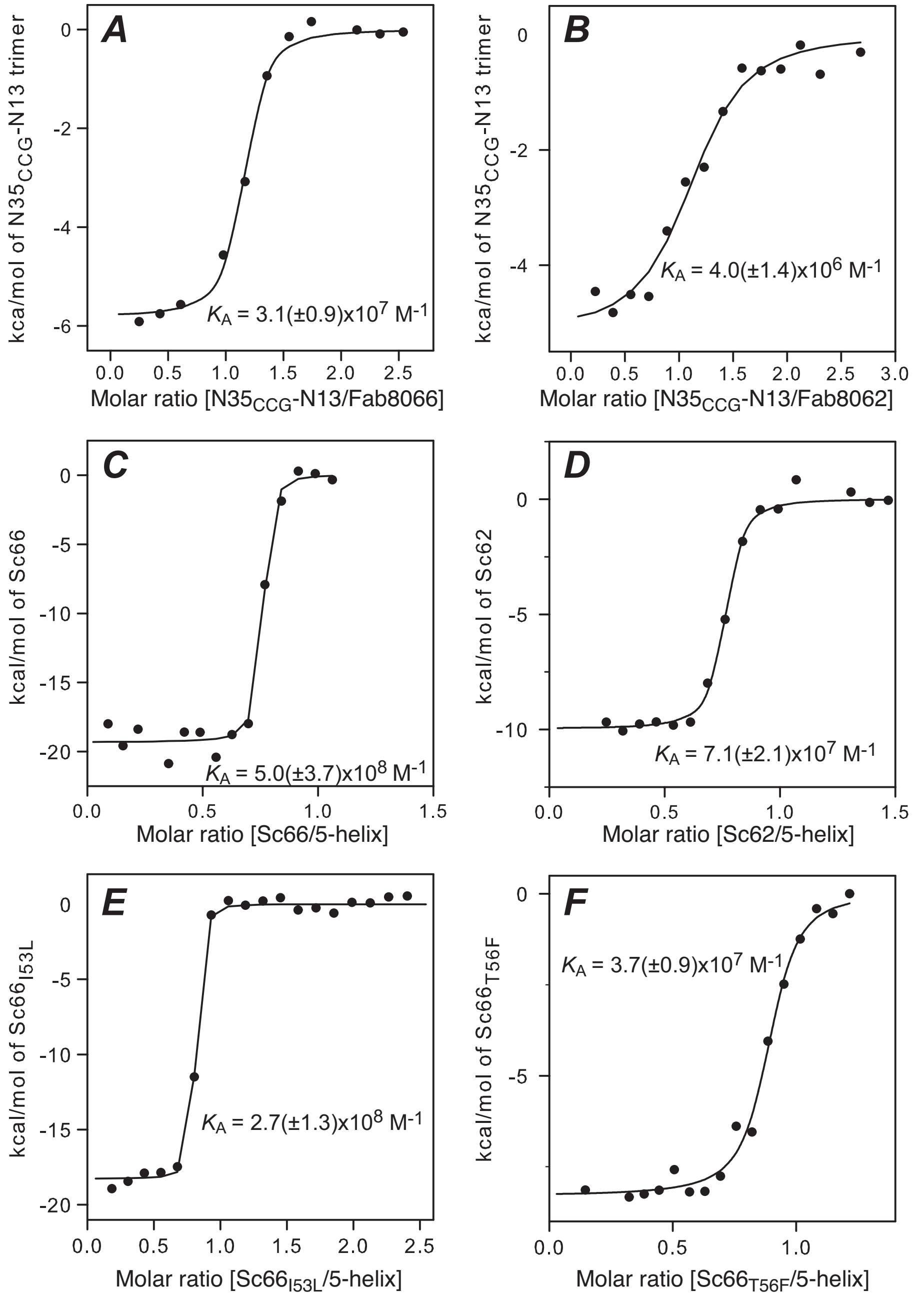

Figure S1. ITC analysis of Fab and ScFv binding to two pre-hairpin intermediate mimetics. Binding of (*A*) Fab8066 and (*B*) Fab8062 to the disulfide-linked trimer N35$_{CCG}$-N13 in 50 mM sodium acetate pH 5.2 buffer (required to maintain the solubility of N35$_{CCG}$-N13). 10 μM Fab was in the cell and N35$_{CCG}$-N13 (~100 μM as monomer) was the titrant. (*C* to *F*) Binding of ScFvs to 5-helix in 10 mM Tris-HCl, pH 7.6, 150 mM NaCl. 5-helix (5-7 μM) in the cell was titrated with the designated ScFv at a concentration of 50-70 μM. HIV-1 neutralizing antibodies are shown in the left-hand panels and non-neutralizing in the right-hand panels. Values of the equilibrium association constant (K_A) are shown in each panel.

A Sc66

```
         10         20         30         40         50         60         70
DIELTQPPSV SVVPGQTARI SCSGDNIPYE YASWYQQKPG QAPVLVIYGD NNRPSGIPER FSGSNSGNTA
                          -----L1-----             -----L2-----
         80         90        100        110        120        130        140
TLTISGTQAE DEADYYCASW DSMTVDGVFG GGTKLTVGGG GSGGGGSGGG GSQVQLVQSG AEVKKPGSSV
                 -----L3----                     3xG4S
        150        160        170        180        190        200        210
                                    L  FA V
KVSCKASGGT FNSYAFSWVR QAPGQGLEWM GSIIPIFGTT NYAQKFQGRV TITADESTST AYMELSSLRS
       -----H1----                -------–H2-------
        220        230        240
EDTAVYYCAR YFDTYNNYGF ANWGQGTLVT VSS
           ------H3-----
```

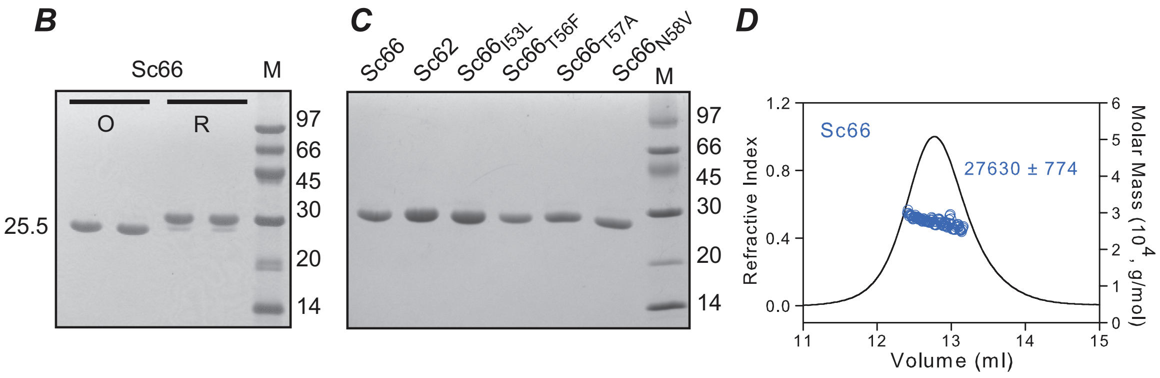

Figure S2. Expression, purification and folding of functional ScFvs. Sc66 and Sc62 are derived from the broadly neutralizing Fab8066 and the non-neutralizing Fab8062, respectively [ref S1]. (*A*) Amino acid sequence of Sc66. Sc62 differs from Sc66 at 4 positions (displayed in green) within the CDR-H2 loop. In the ScFvs, the light (blue) and heavy (orange) variable regions are separated by the 15 residue spacer sequence 3 x (GGGGS) that connects the C-terminus of the light chain variable domain to the N-terminus of the heavy chain variable domain. (*B*) 20% homogeneous SDS-PAGE of Sc66 under oxidizing (lanes O) and reducing (lanes R) conditions shows that complete disulfide bridge formation can be visualized by the faster migration of oxidized Sc66 (lanes O) relative to the reduced sample (lanes R) and can therefore be monitored during ScFv folding. (*C*) 20% homogeneous SDS-PAGE of Sc66, Sc62 and four Sc66 mutants containing point mutations within the CDR-H2. Numbering of the mutations corresponds to their position in the heavy chain variable sequence of Fab8066 [ref S2]. Numbers beside the marker lane (M) in both gel panels denote molecular weights in kDa. (*D*) The mass of Sc66 determined by SEC-MALS corresponds to a monomer (calculated mass, 25,540).

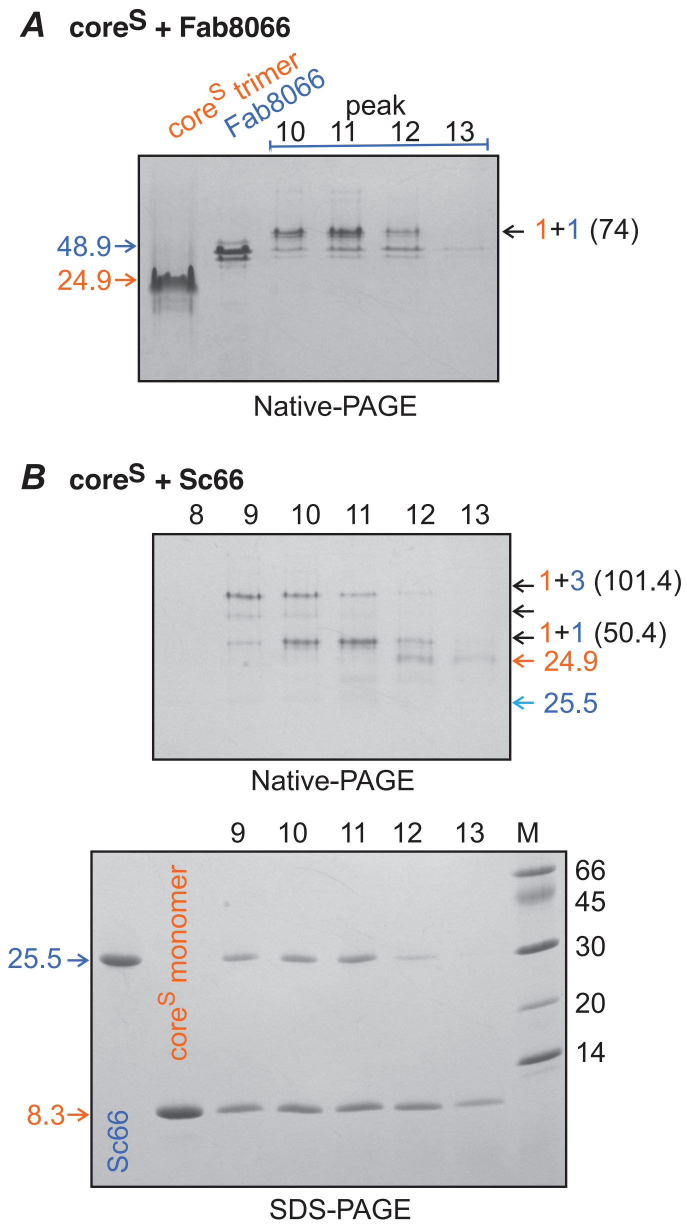

Figure S3. Native- and SDS-PAGE of peak fractions from SEC-MALS analysis of 1:1 mixtures of core^S-Fab8066 and core^S-Sc66 complexes. Gels shown in (*A*) and (*B*) correspond to elution profiles and masses shown for core^S-Fab8066 and core^S-Sc66 in Figs. 3A and B, respectively, in the main text. Numbering of lanes corresponds to elution volume.

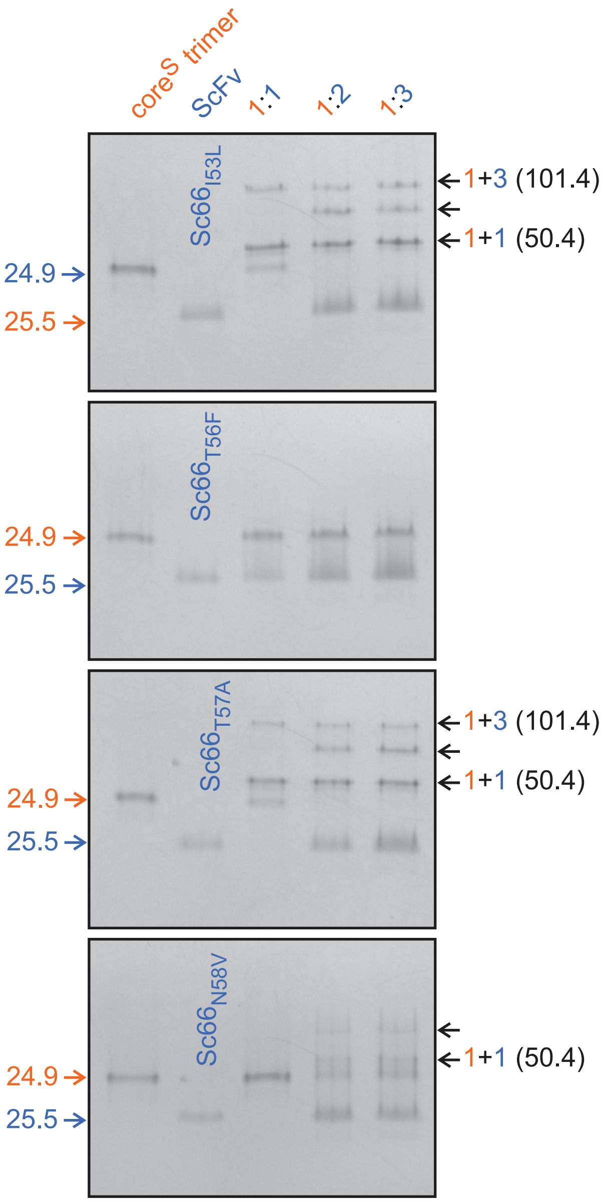

Figure S4. Native-PAGE of core^S-Sc66 single mutant complexes. No binding is observed with Sc66$_{T56F}$, and relatively weak (1:1) binding is seen with Sc66$_{N58V}$. Shifts in the presence of mutants Sc66$_{I53L}$ and Sc66$_{T57A}$ closely resemble those for Sc66. Core^S and ScFv are color coded orange and blue, respectively. Calculated molecular weights of core^S, ScFv and their corresponding complexes are indicated in kDa.

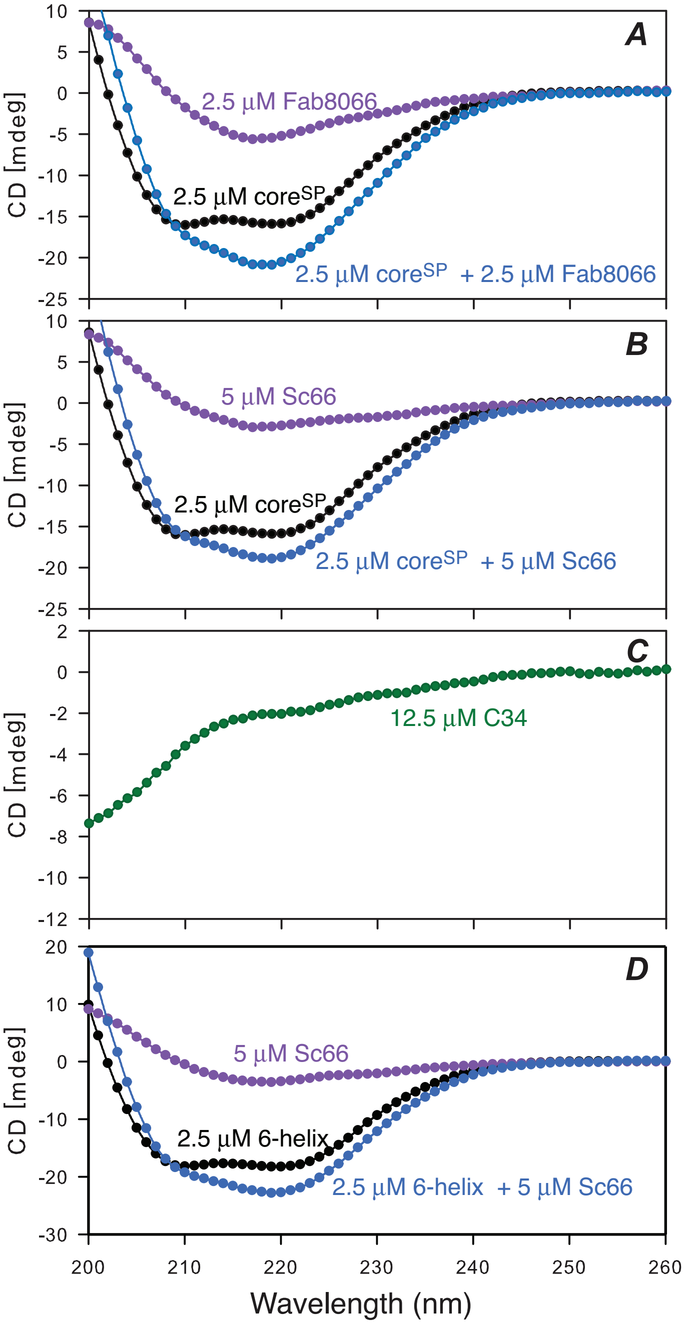

Figure S5. Raw CD data for Fab8066 and Sc66 complexed to the six-helix bundles core^{SP} and 6-helix. (*A*) core^{SP} + Fab8066; (*B*) core^{SP}+Sc66; (*C*) the C-HR peptide C34; (*D*) 6-helix + Sc66.

Supplementary References

S1. Gustchina E, Louis JM, Frisch C, Ylera F, Lechner A, Bewley CA, CloreGM. Affinity maturation by targeted diversification of the CDR-H2 loop of a monoclonal Fab derived from a synthetic naive human antibody library and directed against the internal trimeric coiled-coil of gp41 yields a set of Fabs with improved HIV-1 neutralization potency and breadth. Virology 2009;393:112-119.

S2. Gustchina E, Li M, Louis JM, Anderson DE, Lloyd J, Frisch C, Bewley CA, Gustchina A, Wlodawer A, Clore GM. Structural basis of HIV-1 neutralization by affinity matured Fabs directed against the internal trimeric coiled-coil of gp41. PLoS Pathog 2010;6:e1001182
